# Healthcare workers’ perceptions on collaborative capacity at a Referral Hospital in Malawi

**DOI:** 10.4102/hsag.v26i0.1561

**Published:** 2021-07-30

**Authors:** Tulipoka N. Soko, Diana L. Jere, Lynda L. Wilson

**Affiliations:** 1 Department of Postgraduate Studies, Kamuzu College of Nursing, Blantyre, Malawi; 2 Department of Mental Health, Faculty of Nursing, Kamuzu College of Nursing, Blantyre, Malawi; 3 School of Nursing, University of Alabama at Birmingham, Birmingham, Alabama, United States of America

**Keywords:** collaborative capacity, healthcare workers, interprofessional team, perception, quality of care, patient-centred care

## Abstract

**Background:**

Lack of collaborative capacity results in provision of fragmented health services that do not meet the needs of patients. Collaborative capacity refers to the extent to which providers have influence over other healthcare workers’ decision-making, and can be assessed by measuring perceptions of task interdependence, quality of interaction and collaborative influence. However, each healthcare worker may present differing perceptions that can influence their ability to collaborate effectively during provision of care. No studies that specifically assessed healthcare workers’ perception of collaborative capacity in Malawi were identified.

**Aim:**

To assess the perceptions of healthcare workers regarding collaborative capacity in Malawi.

**Setting:**

The study was conducted at a tertiary public hospital in Blantyre city, Malawi.

**Methods:**

The study employed a quantitative cross-sectional correlational design. The instrument used was a Care Coordination survey that had been used previously in similar studies in the United States of America. Descriptive statistics as well as univariate and multivariate analysis were computed using Statistical Package for Social Science (SPSS) program version 21.0 (IBM, Armonk, NY, USA).

**Results:**

A total of 384 healthcare workers participated in the study, with a response rate of 100%. There were differences in perceptions of collaborative capacity based on the cadre of the respondent (*p* < 0.005). Medical staff reported higher mean scores on quality of interaction (2.94) and collaborative influence (2.65), whereas technical support staff reported the lowest mean scores across all three measures of collaborative capacity (*≤* 2.4).

**Conclusion:**

Differences in perceptions about collaborative capacity suggest the need for interventions to enhance interprofessional collaboration.

**Contribution:**

The study will inform strategies to promote interprofessional collaboration.

## Introduction

Collaboration amongst healthcare workers is increasingly advocated as a means of increasing access to care and improving patient outcomes (Chan & Wood 2010; Smith 2015). Collaborative capacity is a condition needed for coalition, partnership or networks amongst teams in an organisation in order to prioritise critical decisions for patient care (Beier 2014). Collaborative capacity refers to the extent to which healthcare workers have influence over task interdependence, quality of interaction and collaborative influence during provision of patient-centred care. Collaboration further promotes trust and mutual understanding amongst healthcare workers (Weinberg et al. 2011).

Healthcare workers comprise a diverse group of practitioners who deliver quality care to patients in a variety of settings. They include doctors, clinical officers, nurses or midwives, pharmacists, physiotherapists, dentists, radiographers and laboratory technologists (Iwu 2014). All members of the healthcare team have the opportunity to contribute their knowledge, expertise and skills necessary to achieve a holistic management of patients’ health needs (Rose 2011). However, each healthcare worker may present differing perceptions that can influence their ability to collaborate effectively during provision of care (Lankhof 2018). Effective collaboration increases the likelihood that decisions made by healthcare workers are in line with the patients’ needs (Sullivan et al. 2019).

Weinberg et al. (2011) suggested that collaborative capacity includes the following: a team-based approach to care, interdependence, accountability, exchange of knowledge regarding patient information and use of individualised patient care plans to ensure continuity of care. Healthcare workers support each other to develop skills and knowledge about working together and fostering positive attitudes for effective provision of care. Collaborative capacity amongst healthcare workers reduces costs by helping to reduce hospital readmissions and length of hospital stay, and also increases levels of satisfaction amongst both providers and patients (Adebayo & Ilesanmi 2016; Stutsky & Spence Laschinger 2014).

Globally, collaborative capacity amongst health professionals in healthcare settings is limited, resulting in increased numbers of treatment errors, missed opportunities for patient consultation and delayed referral to specialist care (Havens et al. 2010). Martin et al. (2010) suggest that up to 70% of the adverse events in healthcare settings were related to poor collaboration amongst healthcare workers. In developing countries, healthcare services are often fragmented and provision of patient-centred care is limited (Otero et al. 2015).

Challenges in collaboration may be because of the fact that healthcare workers are socialised in their own professions, philosophies, values and theoretical perspectives inherent to their unique professions. As a result, communication amongst health professionals is not emphasised and collaborative capacity is limited (Baker 2010; Needleman et al. 2011). One way to improve patient care is to strengthen relationships and build collaborative capacity amongst healthcare workers (Beier 2014).

Several studies related to collaborative practice have been conducted in Africa (Adebayo & Ilesanmi 2016; Bates 2018; Braun et al. 2011; Otero et al. 2015; Sello & Dambisya 2014). Sello and Dambisya (2014) studied pharmacists in Limpopo province in South Africa and found that 95% of respondents reported that they were not engaged in meetings or ward rounds with other healthcare workers. Similarly, Adebayo and Ilesanmi (2016) conducted a study of physician or nurse collaboration in Nigeria and reported that 80% of the doctors showed poor attitudes to doctor–nurse collaboration; however, 84% of the nurses had a good attitude towards doctor–nurse collaboration.

Braun et al. (2011) conducted a retrospective study on the relationship between coordination of maternal and infant HIV services effects and early infant diagnosis in Lilongwe, Malawi. Findings indicate that a disjointed provision of HIV care services by healthcare workers led to high attrition rates of HIV exposed and infected infants, delayed diagnosis and late initiation of anti-retroviral therapy (ART). The findings also suggest that there was not much sharing of information about patient care amongst healthcare workers.

Weinberg et al. (2011) surveyed 1527 healthcare workers from nine hospitals in New York state to examine collaborative capacity. Their results indicated that physicians reported higher scores on all three components of collaborative capacity (task interdependence, quality of interaction and collaborative influence), and that rehabilitation therapists and paraprofessionals tended to report lower scores. Furthermore, a study reported by Sullivan et al. (2019) found that physicians and nurse practitioners had the highest scores across all three measures of collaborative capacity, whereas direct care workers had the lowest scores.

No studies that specifically evaluated healthcare workers’ perceptions of collaborative capacity in Malawi were identified. It is not clear to what extent healthcare workers perceive collaborative capacity in their work place. Therefore, this research was conducted to assess the perceptions of healthcare workers in this regard. A clear understanding of healthcare workers’ perceptions on collaborative capacity is an important step towards improvement of healthcare workers’ performance and delivery of a patient-centred care. The results of this study will help identify strategies to promote collaborative capacity at the largest tertiary and academic hospital in Malawi.

## Conceptual framework

**FIGURE 1:**
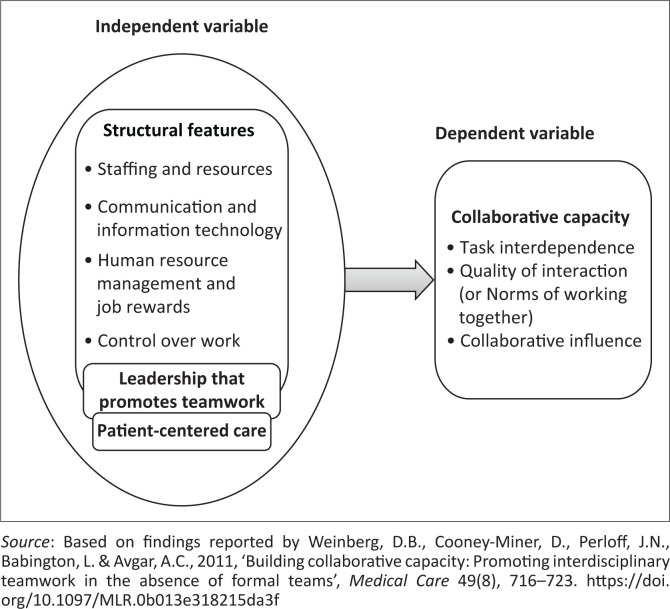
Conceptual framework on collaborative capacity.

The conceptual framework that guided this study was adapted from Weinberg et al. (2011). This framework guided the development of research objectives, organisation of literature, presentation and discussion of results. The conceptual framework proposes that collaborative capacity (a dependent variable) is influenced by structural features that enable team effectiveness, leadership that promotes teamwork and patient-centred care (the independent variables). Figure 1 shows the proposed interaction amongst the study variables. Collaborative capacity can be measured by assessing task interdependence (frequency of interaction and dependence on other healthcare workers for information), quality of interactions (how team members relate to one another) and collaborative influence (egalitarian collaboration amongst team members) (Weinberg et al. 2011).

Weinberg et al. (2011) conceptualised structural features that promote team effectiveness to include four variables: staffing and resources, communication and information technology, human resource management and job rewards, and control over work. Leadership is the capacity to set policy and strategic direction, manage resources and maintain staff commitment to get work done (Bates 2018; Daire, Gilson & Cleary 2014; Weinberg et al. 2011). Patient-centred care refers to a holistic approach aimed at delivery of individualised care to patient and family that addresses their physical, mental, social and self-care needs (Castro et al. 2016).

### Aim

The aim of this study was to assess healthcare workers’ perceptions about collaborative capacity (task interdependence, quality of interaction and collaborative influence) in Blantyre, Malawi.

### Objectives

The objectives of the study were to:

- Describe perceptions about collaborative capacity (task interdependence, quality of interaction and collaborative influence) amongst healthcare workers in Blantyre, Malawi.
- Compare differences in perceptions about collaborative capacity amongst healthcare workers in Blantyre, Malawi, based on the cadre of healthcare workers.

A future publication will report the relationships between measures of collaborative capacity and the independent variables of structural features that promote team effectiveness, leadership and patient-centred care.

## Research method and design

### Study design

This study used a cross-sectional correlational design, utilising a structured questionnaire (the Care Coordination Survey) developed by Weinberg et al. (2011).

### Setting

The study was conducted at a referral hospital in Blantyre, Malawi. The hospital also serves as a training site for health professional students and provides secondary and specialised tertiary level care to both out-patients and in-patients. The hospital was purposively selected because it is the largest government referral and academic hospital in Malawi. This hospital has a total of 49 wards and departments with a capacity of 1400 beds, organised into eight major units according to specialties, namely, Emergency and Trauma, Medical, Surgical, Theatres or Intensive Care, Ambulatory Services and/or Clinics, Obstetrics and Gynaecology, Paediatrics and Technical Support Services.

### Study population and sampling strategy

The study population included all healthcare workers at the hospital who were involved in providing direct patient care. At the time of the study the total number of healthcare workers at the facility was 526: 65 medical doctors, 346 nurses or midwives (registered and technicians), 59 clinical officers, 9 physiotherapists, 14 pharmacists, 25 laboratory technologists and 8 radiographers (HMIS Quarterly Report, 2017).

Using the standard deviation reported in a similar study that was conducted by Weinberg et al. (2011), it was calculated and determined that a sample size of 384 respondents would be needed in order to achieve a margin of error of 0.05 (5%) at the 95% confidence level. Stratified random sampling was used to select the subsamples per cadre. Every member in each stratum, for instance, nurses or midwives or pharmacists, was assigned a number and random numbers from Bluman and Cole (2012) were used to randomly select respondents to be included in the study. A total of 384 healthcare workers were sampled to participate in the study, with a break down per cadre as follows: medical doctors (47), nurses or midwives (252), clinical officers (43), laboratory technologists (18), radiographers (8), pharmacists (10) and physiotherapists (7). Recruitment continued until the desired sample of 384 was achieved, and all who were invited agreed to participate in the study.

### Inclusion criteria

Inclusion criteria for the study were as follows: health worker employees, working in wards or departments during the period of the study, able to read and write in English and willing to consent to participate in the study.

### Data collection

Data were collected by the researcher, assisted by five research assistants. They used a self-administered structured questionnaire adapted from Weinberg et al. (2011) following the receipt of written permission to use the instrument. The questionnaire had four sections with a total of 168 items. The first section included seven questions related to demographic characteristics of participants. The other sections included nine scales with close-ended questions with a five-point Likert scale with responses ranging from *all the time* (4) *to never* (0).

Administration of the questionnaire was arranged at the convenience of the respondents. On the agreed day and time of data collection, the researcher or research assistant met the respondents in a private room free from interruption to promote privacy and confidentiality. Each respondent was given a participant information sheet that explained the study in detail. Additionally, the researcher and research assistants verbally explained the study and reviewed the study information for each respondent. Each respondent was given an opportunity to read through the information sheet and ask questions regarding the protocol before deciding to take part in the study. Once the respondent had read and understood the procedure, the researcher or research assistants obtained written informed consent. The questionnaire was then handed over to the respondent for self-administration. The completed questionnaire was collected by the researcher or research assistant. Data collection was conducted from July to November 2017 and lasted for 20 weeks. Time to complete the questionnaire was on an average about 50 min per participant. No incentives were offered to respondents to complete the questionnaire.

### Validity and reliability

This study adapted and used a Care Coordination survey which was developed and previously validated by Weinberg et al. (2011) in the United States of America. The adapted tool was reviewed by four content experts in Malawi who rated each item on the survey on a four-point scale. A Content Validity Index (CVI) was calculated for each scale by calculating the percentage of experts who rated the scale items as 3 (‘quite relevant’) or 4 (‘highly relevant’). The CVI was 100% on all nine scales. Cronbach’s alpha reliability coefficients for the subscales ranged from 0.70 to 0.99. The researcher trained five research assistants for 2 days on the questionnaire and data collection process in order to maintain internal validity of the study.

### Data analysis

Data were analysed using Statistical Package for Social Science (SPSS) program version 21.0 (IBM, Armonk, NY, USA). Descriptive statistics techniques were used to report means and standard deviations for each of the variables. Kruskal–Wallis tests and post-hoc Bonferroni tests were used to compare the perceptions of collaborative capacity by different cadres of health worker. The statistician who is named in the acknowledgement section assisted with data analysis.

### Ethical considerations

The study was approved by the University of Malawi College of Medicine Research and Ethics Committee (COMREC) (Certificate number: P.02/17/2123). Institutional authorisation to conduct the study was obtained from the hospital director. Information about the study was read to the participants. A written informed consent was obtained from all the participants. Their participation was voluntary and they were told that they could withdraw from the study at any point should they wish so. Confidentiality and anonymity were maintained by collecting limited demographic information and using code numbers.

## Results

A total of 384 healthcare workers participated in the study. The response rate was 100%. The majority of the respondents in the study were women (64.58%; *n* = 248). Most of the respondents had a diploma degree as their highest level of education (42.19%; *n* = 162), followed by a bachelor’s degree (39.32%; *n* = 151). There were more nurse or midwife technicians (40.36%; *n* = 155) compared to other cadres. More than half of the respondents had greater than 5 years of experience in their roles (57.81%; *n* = 222). Together, slightly more than half of the respondents were working in the paediatric (25.52%; *n* = 98) and medical (25.26%; *n* = 97) units (Table 1).

**TABLE 1:** Socio-demographic characteristics of respondents (*n* = 384).

Variable	Frequency	Percentage
**Gender**		
Male	136	35.42
Female	248	64.58
**Highest level of education**		
Certificate	52	13.54
Diploma	162	42.19
Bachelor’s degree	151	39.32
Master’s degree	18	4.69
Doctorate degree	1	0.26
**Years of experience**		
< 1 year	9	2.34
1–2 years	94	24.48
3–4 years	59	15.36
> 5 years	222	57.81
**Roles and/or Cadre**		
Specialists and/or consultants	6	1.56
Registrars	22	5.73
Medical officers	18	4.69
Clinical officers	44	11.46
Registered nurse and/or midwives	96	25.00
Nurse and/or midwife technicians	155	40.36
Physiotherapists	8	2.08
Pharmacists	10	2.60
Laboratory technologists	18	4.69
Radiographers	7	1.82
**Ward and/or Department**		
Accident, Emergency and Trauma (AETC)	9	2.34
Clinics	26	6.77
Medical	97	25.26
Obstetrics and Gynaecology	42	10.68
Paediatric	98	25.52
Surgical	35	9.11
Technical Support	43	11.20
Theatre and/or Intensive Care Unit (ICU)	35	9.11

### Collaborative capacity (task interdependence, quality of interactions and collaborative influence) by role and/or cadre

Table 2 presents the scores on each of the measures of collaborative capacity by role and/or cadre. The overall mean score for respondents on task interdependence was 2.59 (standard deviation [s.d.] = 0.7). Task interdependence was highest amongst physiotherapists (mean [M] = 2.78, s.d. = 0.6) and lowest amongst radiology technologists (M = 2.24, s.d. = 0.2). Quality of interaction was highest (M > 2.7) amongst registrars, clinical officers, specialists, medical officers, nurse midwife technicians and registered nurse midwives and lowest for pharmacists and radiographers. The mean scores for collaborative influence were > 2.5 for all cadres except for pharmacists (M = 1.96, s.d. = 0.9) and radiographers (M = 1.93, s.d. = 0.4). Overall, pharmacists and radiographers reported lowest mean scores (all < 2.4) across all three measures of collaborative capacity (task interdependence, quality of interaction and collaborative influence).

**TABLE 2:** Mean scores on measures of collaborative capacity (task interdependence, quality of interactions and collaborative influence) by role and/or cadre.

Role and/or cadre	Task interdependence		Quality of interactions		Collaborative influence	
	Mean	Standard deviation	Mean	Standard deviation	Mean	Standard deviation
Specialists and/or Consultants	2.47	0.4	2.89	0.6	2.89	0.7
Registrars	2.68	0.6	2.98	0.5	2.63	0.7
Medical officers	2.50	0.5	2.88	0.3	2.62	0.3
Clinical officers	2.59	0.6	2.96	0.5	2.64	0.6
Registered nurses and/or midwives	2.53	0.7	2.75	0.5	2.69	0.7
Nurse and/or midwife technicians	2.64	0.8	2.82	0.6	2.58	0.8
Physiotherapists	2.78	0.6	2.68	0.6	2.50	0.7
Pharmacists	2.40	0.8	2.22	0.3	1.96	0.9
Laboratory technologists	2.76	1.1	2.60	0.6	2.08	1.0
Radiographers	2.24	0.2	2.34	0.4	1.93	0.4
Overall sample	2.59	0.7	2.79	0.5	2.57	0.7

Table 3 presents the results of non-parametric Kruskal–Wallis tests which were performed to establish whether collaborative capacity differed significantly between respondents based on their cadre. The results show that all three components of collaborative capacity varied significantly amongst roles and/or cadres: task interdependence (χ^2^ = 17.6, *p* = 0.046), quality of interaction (χ^2^ = 29.3, *p* = 0.001) and collaborative influence (χ^2^ = 21.5, *p* = 0.011).

**TABLE 3:** Results of Kruskal–Wallis equality of populations rank test for collaborative capacity (task interdependence, quality of interactions and collaborative influence) by role and/or cadre (*n* = 384).

Role and/or cadre	Task interdependence		Quality of interactions		Collaborative influence	
	*n*	Rank sum	*n*	Rank sum	*n*	Rank sum
Specialists and/or consultants	6	1028	6	1231	6	1419.5
Registrars	22	4536	22	5058.5	22	4555.5
Medical officers	18	3151.5	18	3793	18	3530.5
Clinical officers	44	8503	44	10096.5	44	8679
Registered nurse and/or midwives	96	17 466	96	17632.5	96	20013.5
Nurse and/or midwife technicians	155	30 995	154	30289.5	155	30 236
Physiotherapists	8	1754.5	8	1377	8	1449
Pharmacists	10	1590.5	10	724.5	10	1180.5
Laboratory technologists	18	3998	18	2683.5	18	2300.5
Radiographers	7	897	7	650	7	556
Degrees of freedom	-	9	-	9	-	9
Chi-square	-	17.6	-	29.3	-	21.5
Probability	-	0.046	-	0.001	-	0.011

In order to further examine differences in the three measures of collaborative capacity by cadre, the individual cadres were combined into three groups: medical staff (including specialists and/or consultants, registrars, medical officers and clinical officer), nursing and/or midwifery staff (including registered nurses and/or midwives, and nurse and/or midwife technicians) and technical support staff (including pharmacists, physiotherapists, laboratory technologists and radiographers). Table 4 shows the results of comparisons amongst these three groups.

**TABLE 4:** Means of collaborative capacity (task interdependence, quality of interactions and collaborative influence) based on collapsed categories of cadre (medical staff, nursing and/or midwifery staff and technical support staff).

Specialisation	Task interdependence		Quality of interactions		Collaborative influence	
	Mean	Standard deviation	Mean	Standard deviation	Mean	Standard deviation
Medical staff	2.59	0.6	2.94	0.5	2.65	0.6
Nursing and/or midwifery staff	2.59	0.8	2.79	0.6	2.62	0.8
Technical support staff	2.59	0.8	2.49	0.5	2.11	0.8
Overall sample	2.59	0.7	2.79	0.5	2.57	0.8

The mean scores for task interdependence were similar (2.59 with s.d. of 0.6–0.8) for all three groups. Mean scores for quality of interactions ranged between (M = 2.94, s.d. = 0.5) and (M = 2.49, s.d. = 0.5), with the highest scores reported by medical staff (M = 2.94, s.d. = 0.5), slightly lower scores reported by nursing/midwifery staff (M = 2.79, s.d. = 0.6) and lowest scores reported by technical support staff (M = 2.49, s.d. = 0.5). Mean scores for collaborative influence indicate that medical staff and nursing and/or midwifery staff had higher mean scores compared to technical support staff. Overall, medical staff had the highest mean on quality of interactions and collaborative influence, and technical support staff reported the lowest mean scores on these variables.

Non-parametric Kruskal–Wallis tests were conducted to establish whether collaborative capacity differed significantly amongst the three groups of respondents based on the collapsed categories. The results presented in Table 5 show that all three components of collaborative capacity varied significantly between cadres as follows: task interdependence (χ^2^ = 9.9, *p* = 0.02), quality of interaction (χ^2^ = 22.7, *p* = 0.001) and collaborative influence (χ^2^ = 16.6, *p* = 0.001).

**TABLE 5:** Results of Kruskal–Wallis equality of populations rank test for collaborative capacity by specialisation (*n* = 384).

Specialisation	Task interdependence		Quality of interaction		Collaborative influence	
	*n*	Rank sum	*n*	Rank sum	*n*	Rank sum
Medical staff	90	17 219	90	20 179	90	18184.5
Nursing and/or midwifery staff	251	48 461	251	47 922	251	50249.5
Technical support staff	43	8240	43	5435	43	5486
Degrees of freedom	-	2	-	2	-	2
Chi-square	-	9.9	-	22.7	-	16.6
Probability	-	0.020	-	0.001	-w	0.001

Post hoc Bonferroni analyses were conducted to further explore these differences. For the measure of collaborative influence, there was a significant difference between medical staff and technical support staff (*p* < 0.001), and between nurses and/or midwifery staff and technical support staff (*p* < 0.001). For the measure of quality of interactions, there was a significant difference between medical staff and technical support staff (*p* < 0.001) and between nursing/midwifery staff and technical support staff (*p* < 0.001). For the measure of task interdependence, there was a significant difference between medical staff and nursing and/or midwifery staff (*p* < 0.001) and between nursing and/or midwifery staff and technical support staff (*p* < 0.006).

This study was a replication of two other studies conducted in the USA by Weinberg et al. (2011) and Sullivan et al. (2019). Weinberg et al. (2011) studied 1527 healthcare workers in nine hospitals and seven healthcare systems in upstate New York, USA. Sullivan et al. (2019) studied 723 healthcare workers in 20 community living centres managed by the Veterans Administration in the USA.

Table 6 compares the means and standard deviations of the major study variables across the three studies (Sullivan et al. 2019; Weinberg et al. 2011 and the present study). All three studies used the same measures and a five-point Likert scale; however, Sullivan et al. (2019) used a range of 1–5, whereas Weinberg et al. (2011) and the present study used a range of 0–4. Thus, the means reported by Sullivan et al. (2019) need to be adjusted by lowering them with a 1-point in order to compare them with the means reported by Weinberg et al. (2011) and the present study.

**TABLE 6:** Comparison between Weinberg et al. (2011) and Sullivan et al. (2019) studies and the present study.

Variable name	Findings from Sullivan et al. (2019)†		Findings from Weinberg et al. (2011)		Findings from the present study	
	Mean	Standard deviation	Mean	Standard deviation	Mean	Standard deviation
Task interdependence	3.59	0.8	2.61	0.7	2.59	0.7
Quality of interactions	3.85	0.7	2.91	0.5	2.79	0.5
Collaborative influence	3.30	0.9	2.35	0.9	2.57	0.8

† , Sullivan et al. (2019) used a Likert scale with values ranging from 1 to 5, whereas Weinberg et al. (2011) and the present study used scales with values ranging from 0 to 4, and thus the Sullivan et al. means are higher. These mean scores should be decreased by 1 point in order to compare them with the other two studies.

The specific cadres of healthcare worker differed in the three studies. Weinberg et al. (2011) compared physicians, nurse practitioners or physician assistants, rehabilitation therapists, case managers or social workers, registered nurses, nurses’ aides, clerks and secretaries. The cadres in the Sullivan et al. (2019) study included physicians, nurse practitioners, rehabilitation therapists, nurses, social workers and/or case managers, direct care workers, nutritionists, pharmacists, psychologists and ‘other’. The cadres in the current study included specialists and/or consultants, registrars, clinical officers, medical officers, registered nurses and/or midwives, nurse and/or midwife technicians, physiotherapists, pharmacists, laboratory technologists and radiographers.

For the purpose of comparison, a difference of 0.5 in the means was considered suggestive of a difference in the means reported across the three studies. The mean scores reported by Sullivan et al. (2019) were decreased by 1.0 for this comparison, as those researchers used a scale from 1 to 5 rather than the 0–4 scale used by Weinberg et al. and in the present study. The study findings revealed that scores on task interdependence, quality of interactions and collaborative influence were similar in the three studies. The standard deviations for the variables across the three studies ranged from 0.49 to 0.86.

## Discussion

Mean scores on task interdependence in the current study were significantly different based on analyses of each individual cadre as well as the combined groups. The scores were highest amongst physiotherapists, laboratory technologists, registrars, nurse and/or midwives technicians, clinical officers, registered nurse and/or midwives and medical officers, respectively, and lowest amongst radiographers. When the cadres were combined into three groups (medical staff, nursing and/or midwifery staff and technical support staff), the mean scores on task interdependence were similar across all groups, although the results of Kruskal–Wallis tests indicated significant differences amongst the three cadres. This finding is similar to the findings reported by Weinberg et al. (2011) who stated that doctors, nurses, social workers, physician assistants and case managers had significantly higher levels of task interdependence than did rehabilitation specialists, nurses’ aides and patient care technicians. Similarly, Sullivan et al. (2019) stated that doctors, nurse practitioners, nurses and social workers had higher scores on task interdependence compared to the other cadres studied.

Mean scores on quality of interaction in the current study were significantly different based on analyses of each individual cadre as well as the combined groups. Quality of interaction scores were highest amongst medical staff, slightly lower amongst nursing and/or midwifery staff and lowest amongst support staff. This finding is slightly different from the findings reported by Weinberg et al. (2011) who stated that doctors, nurse practitioners, case managers and social workers had higher quality of interaction than nurses or rehabilitation therapists. Sullivan et al. (2019) found that highest quality of interaction mean scores were reported by physicians, nurse practitioners and/or nurse managers, social workers, nutritionists, psychologists and rehabilitation specialists, with lower scores reported by nurses and lowest mean scores reported by direct care workers.

Mean scores on collaborative influence in the present study were also significantly different based on the analyses of each individual cadre as well as the three collapsed groups, and indicated that the mean scores were higher for medical staff and nursing and/or midwifery staff and lowest for technical support staff. This finding is similar to the findings of Weinberg et al. (2011) who reported that doctors, physician assistants and nurse practitioners reported higher levels of collaborative influence than any other group. Sullivan et al. (2019) also found that mean scores on all measures of collaborative capacity differed by the cadre of the worker. Physicians, nurse practitioners and nurse managers reported significantly higher scores than did direct care workers such as licensed practical nurses or nursing assistants.

The findings from the two studies in the USA and the current study in Malawi suggested that higher status occupations such as physicians, nurse practitioners and physician assistants reported higher levels of task interdependence, quality of interactions and collaborative influence, compared with more technical occupational groups who might be considered to have a lower status. The findings that the technical support staff in Malawi reported lower mean score levels in all three measures of collaborative capacity might be explained by the fact that these healthcare workers mostly work in their individual departments rather than in hospital wards, and are usually not involved in ward rounds or ward meetings. They only provide support to other healthcare workers or consult upon request. Medical staff members tend to be more engaged in patient care in the wards of the study hospital which is a referral hospital. They examine, diagnose and prescribe treatment for patients with complex conditions (Government of Malawi 2018).

The significant difference in perceptions about collaborative capacity according to roles and/or cadres of healthcare workers leads to the conclusion that healthcare workers perceived collaborative capacity differently. This could be as a result of their professional socialisation during pre-service education and educational level (Baker 2010; Lam et al. 2015; Price, Doucet & Hall 2014). In agreement, Price et al. (2014) determined that during pre-service education, students are ‘socialised’ to adopt a specific discipline-based vision of the services which they will be offering after they qualify, leading to poor conceptualisation of collaborative practice.

The lower mean scores by the technical support staff is a challenge as this could be explained that the cadre may not effectively engage with other healthcare workers on issues affecting patient care. Similarly, many studies have identified the negative impact on patient care when all cadres do not collaborate effectively (Beier 2014; Kelly et al. 2013). In order to effectively collaborate with each other, there is a need to strengthen interprofessional communication amongst healthcare workers as this would promote mutual respect and overall positive outcomes on patient care (World Health Organization 2010). Baker et al. (2011) suggested on the need of effective leadership that promotes interdependent behaviour like cooperation, knowledge sharing and mutual assistance on tasks to improve collaboration across members of the healthcare team.

Although healthcare workers must consider the skills and qualities of other professionals during communication, there is a need for change in their socialisation. Lam et al. (2015) reported that socialisation could begin with the adoption of interprofessional education during pre-service education in health training institutions. During interprofessional education, ‘students from two or more professions learn about, from and with each other to enable effective collaboration and improve health outcome’ (World Health Organization 2010).

In addition, the differences in the level of education influence the way healthcare workers perform and communicate in the health facility (Aiken et al. 2014; Baker 2010). How each healthcare worker contributes during provision of care is based on the knowledge, skills and expertise they possess. A high level of educational qualification is associated with acquisition of higher knowledge, skills and attitudes necessary in managing patients with complex conditions, and therefore reducing errors and promoting positive health outcomes. Results of a study by Matziou et al. (2014) showed that a university degree and postgraduate studies were significant to promote interprofessional communication and collaborative capacity.

## Limitations of the study

Although the findings of this study contribute to an understanding of collaborative capacity in an African setting, there are limitations that must be acknowledged. This study used a cross-sectional design, and thus the findings reflect only the specific time when the research was conducted. Lastly, the sample included only healthcare workers from a government tertiary health facility. Limited financial resources precluded the inclusion of additional hospitals in this study. The results may only be generalisable to the population at the hospital where the study was conducted but would not be extrapolated to other facilities in Malawi, including district, Christian Health Association of Malawi (CHAM) and private hospitals. However, although the results may limit the generalisability of the study, the study site was the largest public referral hospital and hence some lessons may be generalisable to similar settings.

## Implications for education, practice and research

Findings of differences in perceptions of collaborative capacity based on the cadre of healthcare workers have implications on education and practice. There is a need for both pre-service and in-service education to promote interprofessional team work amongst different cadres of healthcare workers through provision of orientation sessions for new healthcare workers on Interprofessional Collaborative Practice competencies in order to promote positive perceptions about collaborative capacity and quality healthcare.

There is a need for further research in a variety of settings in order to better understand further differences in perceptions about collaborative capacity. Although the study instrument had adequate internal consistency reliability, there is a need for further research to confirm the validity of the measure across different settings. The findings of this study can be used to guide educators, practitioners, researchers, policymakers and other stakeholders in developing policies, guidelines, protocols and standards necessary to promote positive perceptions about collaborative capacity.

## Conclusion

This study highlights perceptions of healthcare workers about collaborative capacity. There were significant differences in the perceptions according to cadres of healthcare workers. It is envisaged that the differences in perceptions about collaborative capacity suggest the need for policymakers, practitioners, educators and researchers to promote interventions for interprofessional collaboration and quality patient care.
